# MiR-200c-3p Contrasts PD-L1 Induction by Combinatorial Therapies and Slows Proliferation of Epithelial Ovarian Cancer through Downregulation of β-Catenin and c-Myc

**DOI:** 10.3390/cells10030519

**Published:** 2021-03-01

**Authors:** Eleni Anastasiadou, Elena Messina, Tiziana Sanavia, Lucia Mundo, Federica Farinella, Stefano Lazzi, Francesca Megiorni, Simona Ceccarelli, Paola Pontecorvi, Francesco Marampon, Cira Rosaria Tiziana Di Gioia, Giorgia Perniola, Pierluigi Benedetti Panici, Lorenzo Leoncini, Pankaj Trivedi, Andrea Lenzi, Cinzia Marchese

**Affiliations:** 1 Department of Experimental Medicine, Sapienza University of Rome, 00161 Rome, Italy; elena.messina@uniroma1.it (E.M.); federica.farinella1@gmail.com (F.F.); francesca.megiorni@uniroma1.it (F.M.); simona.ceccarelli@uniroma1.it (S.C.); paola.pontecorvi@uniroma1.it (P.P.); pankaj.trivedi@uniroma1.it (P.T.); andrea.lenzi@uniroma1.it (A.L.); cinzia.marchese@uniroma1.it (C.M.); 2 Department of Medical Sciences, University of Torino, 10126 Torino, Italy; tiziana.sanavia@unito.it; 3 Department of Medical Biotechnology, Section of Pathology, University of Siena, 53100 Siena, Italy; lucia.mundo.ml@gmail.com (L.M.); lazzi2@unisi.it (S.L.); lorenzo.leoncini@dbm.unisi.it (L.L.); 4 Health Research Institute, University of Limerick, Limerick V94 T9PX, Ireland; 5 Department of Radiotherapy, Policlinico Umberto I, Sapienza University of Rome, 00161 Rome, Italy; francesco.marampon@uniroma1.it; 6 Department of Radiological, Oncological and Pathological Sciences, Sapienza University of Rome, 00161 Rome, Italy; cira.digioia@uniroma1.it; 7 Department of Gynecological-Obstetric Sciences and Urological Sciences, Sapienza University of Rome, 00161 Rome, Italy; giorgia.perniola@uniroma1.it (G.P.); pierluigi.benedettipanici@uniroma1.it (P.B.P.)

**Keywords:** epithelial ovarian cancer, immune checkpoints, PARPi, ionizing radiation, miRNA-based therapy

## Abstract

Conventional/targeted chemotherapies and ionizing radiation (IR) are being used both as monotherapies and in combination for the treatment of epithelial ovarian cancer (EOC). Several studies show that these therapies might favor oncogenic signaling and impede anti-tumor responses. MiR-200c is considered a master regulator of EOC-related oncogenes. In this study, we sought to investigate if chemotherapy and IR could influence the expression of miR-200c-3p and its target genes, like the immune checkpoint PD-L1 and other oncogenes in a cohort of EOC patients’ biopsies. Indeed, PD-L1 expression was induced, while miR-200c-3p was significantly reduced in these biopsies post-therapy. The effect of miR-200c-3p target genes was assessed in miR-200c transfected SKOV3 cells untreated and treated with olaparib and IR alone. Under all experimental conditions, miR-200c-3p concomitantly reduced PD-L1, c-Myc and β-catenin expression and sensitized ovarian cancer cells to olaparib and irradiation. In silico analyses further confirmed the anti-correlation between miR-200c-3p with c-Myc and β-catenin in 46 OC cell lines and showed that a higher miR-200c-3p expression associates with a less tumorigenic microenvironment. These findings provide new insights into how miR-200c-3p could be used to hold in check the adverse effects of conventional chemotherapy, targeted therapy and radiation therapy, and offer a novel therapeutic strategy for EOC.

## 1. Introduction

Ovarian cancer (OC) is the fifth most common cancer worldwide. It includes highly heterogeneous subgroups based on the primary site of origin, and the clinicopathologic, immunohistochemical, and molecular profiles [1]. Its heterogeneity is represented by epithelial cancer cells, which belong to four main histological subtypes: serous, endometrioid, mucinous and clear cells [2]. Every histological subtype is characterized by a different degree of differentiation starting from grade 1 (well differentiated) to grade 3 (poorly differentiated) and, based on the aggressiveness and invasiveness, each is divided into low-grade type 1 and high-grade type 2. According to the International Federation of Gynecology and Obstetrics (FIGO), there are five stages of ovarian cancer, from stage 0, with no evidence of primary tumor, to stage IV, with severe metastases in several organs [3]. Approximately 65% of ovarian neoplasms are epithelial ovarian cancers (EOC), which include serous adenocarcinoma and high-grade serous ovarian cancer (HGSOC) subtypes [4]. The mainstay therapy is de-bulking surgery followed by adjuvant, platinum containing chemotherapy. Despite the first-line therapy, the prognosis of EOC patients in terms of five-year survival rate decreases from 90% at stage I to 20% at stage III-IV of the disease. The high mortality rate depends on the obsolete existence of early-stage screening and development of resistance to platinum-based chemotherapy in almost 80% of the patients [5,6]. To overcome therapeutic failures, targeted therapies including the recently FDA approved poly(ADP-ribose) polymerase inhibitors (PARPi), radiotherapy and immunotherapy have been developed [7,8].

A PARPi, such as olaparib, induces synthetic lethality in OC cells with mutated breast cancer 1 or 2 (BRCA1/2) genes and homologous recombination deficient (HRD) mechanisms by selectively targeting tumor cells that fail to repair DNA double strand breaks (DSBs) [9]. When PARPi is combined with chemotherapy or IR, it may enhance DNA damage in advanced EOC patients carrying mutated or wild type (wt) BRCA1/2, although it is less effective in most BRCA1/2-proficient OC patients [10,11].

Chemotherapy, PARPi and radiotherapy, as monotherapies or combined, might either enhance cytotoxic T lymphocytes against tumor cells or induce immunosuppressive effects through up-regulation of PD-L1 (also known as CD274) [12,13]. High PD-L1 expression in EOC cells has been associated with poor prognosis and antibodies against PD-1/PD-L1 have been used as a therapeutic option in patients with advanced EOC [14]. Intravenous administration of anti PD-1 nivolumab antibody showed a 10–20% overall response rate (ORR) in platinum-resistant and advanced OC patients [15]. A combinatorial therapeutic approach of PARPi and anti-PD-L1 antibodies has shown promising antitumor activity and increased the ORR further in platinum-resistant wtBRCA and non-HRD OC patients [16]. However, additional validation of the synergistic combination of these targeted agents is needed.

MicroRNAs (miRNAs) are becoming important diagnostic and prognostic markers in various cancer types, including OC [17] and they have also been proposed as therapeutic agents against cancer [18,19]. MiRNAs are highly conserved small non-coding RNAs of 20–22 nucleotides (nt) that inhibit gene expression through base-pairing between the seed sequence on the 5′-end of miRNAs and the 3′UTR of mRNA target genes. This binding destabilizes mRNA target genes and/or inhibits mRNA translation [20]. This mechanism of miRNA–mRNA interaction occurs in physiological as well as pathological conditions, such as cancer [21,22]. In OC, the miR-200 family is considered a master regulator of OC-related genes, which inhibit cell migration. This family consists of five members: miR-200a, miR-200b, miR-200c, miR-429 and miR-141, clustered in two different chromosomal locations: miR-200a, miR-200b and miR-429, on chromosome 1p36, and miR-200c and miR-141, on chromosome 12p13 [23]. They usually function as tumor suppressors in various types of cancer. Loss of miR-200 family members is associated with lack of E-cadherin expression in breast and ovarian epithelial cells. Over-expression of miR-200 in these tumors is able to reconstitute E-cadherin expression and promote mesenchymal–epithelial transition (MET) [24]. The transcription repressors of E-cadherin, ZEB1 and ZEB2, which both promote the epithelial–mesenchymal transition (EMT), are known targets of the miR-200 family [25]. Within the miR-200 family, miR-200c regulates EMT-mediated tumor metastasis [26] and fibroblast growth factor receptor (FGFR)-mediated epithelial proliferation [27,28]. Moreover, miR-200c represses the Wnt pathway by targeting β-catenin in breast cancer [29,30]. High expression of β-catenin is associated with resistance to platinum-based chemotherapy in EOC patients [31]. Interestingly, high expression of miR-200c in OC patients was associated with improved overall survival (OS) and progression-free survival (PFS) [17]. Mounting evidence suggests a role for miR-200c and miR-34a in regulating PD-L1 expression in other types of cancer [32,33,34,35]. However, the effect of chemotherapy, PARPi and radiation therapy on miR-200c and consequently on PD-L1 expression has not yet been thoroughly investigated in EOC.

In the present study, we sought to examine in clinical samples of EOC whether the first line chemotherapy might have any impact on PD-L1 expression and if miR-200c-3p is involved in this. The regulation of PD-L1, c-Myc and β-catenin by miR-200c alone or in combination with olaparib and IR was further investigated in SKOV3 cells.

## 2. Materials and Methods

### 2.1. Biopsies and Cell Lines

Laparoscopic ovarian biopsies, before and after chemotherapy, were obtained from a cohort of five EOC patients, classified as HGSOC (Table 1). All five patients involved in this study gave their written consent. The study design was approved by the Ethics Committee of Policlinico Umberto I Hospital, C.E. Ref: 1454/24.07.08, Prot. no. 702/08 (Rome, Italy). Biopsies were snap-frozen and stored at −80 °C. To identify differences in gene expression by chemotherapy, we used biopsies from patients before chemotherapy treatment as controls. After de-bulking surgery and biopsies, tumor cells were classified as Grade 3, known also as high grade ovarian cancer cells. SKOV3 is a wtBRCA1 gene-carrying ovarian serous adenocarcinoma cell line and was purchased from ATCC^®^ (HTB-77™). With one exception, the patients did not present with BRCA1/2 mutations. The cells were cultured in RPMI-1640, (Sigma-Aldrich; Merck Life Science S.r.l., Milan, Italy, Cat. n.R0883), supplemented with 10% FBS (Corning™; Cat.n.15377636), and 100 U/mL penicillin, 100 µg/mL streptomycin, and 29.2 mg/mL of l-glutamine (Gibco™ Penicillin-Streptomycin-Glutamine (100×) Cat.n.10378-016).

### 2.2. RNA Extraction from EOC Biopsies and Cell Lines

Biopsies were collected and stored at −80 °C in RNAlater™ Stabilization Solution (Invitrogen, Milan, Italy; Cat. n.AM7021). Total RNA from OC biopsies was extracted with Qiagen TissueLyser II using TRIzol™ reagent (Invitrogen, Milan, Italy; Cat. n.15596026). Further details of RNA extraction are in Supplementary Materials and methods. A 5 mm^2^ piece from each biopsy was processed through three cycles of sonication of 2 min each at 30 Hrtz in 1 mL TRIzol™ (Invitrogen). RNA samples obtained with phenol-chloroform extraction were quantified using a MaestroNano micro-scale spectrophotometer (MaestroGen Inc., Hsinchu city, Taiwan) and evaluated for quality by a run on 1% agarose gel.

### 2.3. Real-Time qPCR

Retro-transcription of 1 μg RNA from biopsies and cell lines was performed in a BioRad Mycycler Thermal Cycler machine, with miScript II RT Kit (QIAGEN S.r.l., Milan, Italy; Cat. n.218161) according to the manufacturer’s instructions and using HiFlex buffer, for parallel quantification of miRNAs and mRNAs from the same cDNA. One microliter of cDNAs was used as a template for qPCR with the miScript SYBR Green PCR Kit (QIAGEN; Cat. n.218073). RNA from 3 × 10^6^ SKOV3 cells were also extracted with 1 mL TRIzol™, according to the manufacturer’s instructions. All of the quantitative PCRs were performed in an Applied Biosystems\StepOne Software v2.2.2 QPCR machine. The fold change of PD-L1 and miR-200c-3p was calculated by the 2^−ΔΔCt^ formula and the results were statistically analyzed by PRISM7, using two-tailed unpaired t test. Additional details regarding the QuantiTect Primer Assays (QIAGEN) and KiCqStart™ (Sigma-Aldrich) pre-designed primers and qPCR conditions can be found as Supplementary Materials.

### 2.4. Transfections

SKOV3 cells (0.5 × 10^6^) were placed in a 12-well cell culture plate. After 24 h, they were transfected with 1 μg of pCMVmiR-200c carrying the miR-200c precursor (pre-miR-200c) (OriGene Technologies, Inc., Rockville, USA; Cat.n.SC400258) and the corresponding empty vector, pCMV (OriGene; PCMVMIR). The plasmids were transfected with Lipofectamine 3000 (ThermoFisher Scientific, Waltham, MA, USA; Cat. n.L3000008) following the manufacturer’s instructions. After 48 h post transfection, the cells were resuspended in fresh culture medium supplemented with 0.5 mg/mL G418 (Roche Diagnostics, Mannheim, Germany; Cat. n.04 727 878 001) and distributed in a 96 well plate. After two weeks, G418 resistant, pCMV vector and miR-200c-expressing clones started to grow. A previously established reporter system of PD-L1 3′UTR psiCHECK™-2 Vector (Promega Corporation, Madison, WI, USA; Cat. n.C8021) was transiently transfected into SKOV3 pCMV Vector and SKOV3 pCMVmiR-200c carrying SKOV3 cell lines [33]. Approximately 4 × 10^3^ cells per well were distributed in 96-well plates, one day before transfections and in sixplicates. Forty nanograms of PD-L1 3′UTR Luciferase reporter construct per well was transfected using DharmaFECT Duo Transfection Reagent (Dharmacon, Horizon a PerkinElmer company, Diatech, Jesi, Italy; Cat. n.T-2010-02), according to the manufacturers’ instructions. Luciferase activity of PD-L1 3′UTR was measured in miR-200c-SKOV3 cells 48 h post-transfection using GloMax^®^ Explorer Multimode Microplate Reader (Promega). Furthermore, 1 × 10^6^ SKOV3 cells were transiently transfected in a 6 well plate and in triplicates with 40 nM mimic miR-200c-3p oligonucleotide (MISSION^®^, Sigma-Aldrich; Cat. n.HMI0354-5NMOL) and the same amount of negative control 1 based upon an *Arabidopsis thaliana* sequence (MISSION^®^, Sigma-Aldrich; Cat. n.HMC0002). After 48 h, the cells were collected for RNA and protein extraction. In parallel, 4 × 10^3^ SKOV3 cells per well were seeded in sixplicates in a 96 well plate. The next day, the cells were co-transfected with 40 nM mimic miR-200c-3p oligonucleotide (MISSION^®^, Sigma-Aldrich; Cat. n.HMI0354-5NMOL) and its negative control 1, along with 40 ng PD-L1 3′UTR psiCHECK™-2 Vector, using DharmaFECT Duo Transfection Reagent (Dharmacon; Cat. n.T-2010-02). Luciferase activity was measured 48 h post transfection, using GloMax^®^ Explorer. Luciferase assay was repeated twice and in sixplicates. Ratios of firefly/renilla were calculated and *p*-values were calculated by two-tailed unpaired *t*-test.

### 2.5. Cell Treatments

SKOV3 cells, (1 × 10^6^), stably transfected with pCMV Vector and miR-200c were plated in T25 flasks. The next day, the cells were treated with 1.5 μM and 5 μM olaparib (AZD-2281, Selleckchem, Suffolk, UK). Dimethyl sulfoxide (DMSO) (Sigma-Aldrich; Cat. n.D 2650), 0.1 % (*v*/*v*), was used as a drug vehicle. At 48 h post-olaparib treatment, the cells were irradiated using an ONCOR Impression Linear Accelerator (Siemens Medical Solutions USA, Inc., Concord, CA, USA) at a dose rate of 4 Gray (Gy) (416 UM/min) in a field of 40 × 40. Twenty-four hours later, the treated cells were prepared for the clonogenic assay and total or cytoplasmic and nuclear protein extractions.

### 2.6. Colony Formation Assay

At 72 h post-olaparib treatment and 24 h post-IR, 2 × 10^3^ cells (SKOV3 pCMV Vector and SKOV3 miR-200c) were placed in triplicates in 6-well plates for a couple of weeks to estimate colony formation. Untreated cells (DMSO-treated), were used as controls. During the clonogenic assay, the cell culture medium was replenished every three days. Clonogenic assays were repeated at least three times. Further details are in Supplementary Materials and Methods.

### 2.7. Immunoblotting

Three million pCMV Vector or miR-200c transfected SKOV3 cells were collected and lysed in RIPA lysis buffer. Thirty micrograms of protein of each sample was loaded on 10% polyacrylamide gels and run for 1.5 h at 120 V. The following antibodies were used: PD-L1 (E1L3N) (Cell Signaling Technology, Danvers, MA, USA; Cat. n.13684), β-catenin (E-5) (Santa Cruz Biotechnology, Inc., Heidelberg, Germany; Cat. n.sc-7963), and c-Myc (D84C12) (Cell signaling; Cat. n.5605). β-actin (C4) (Santa Cruz; Cat. n.sc-47778) and Lamin B1 (C-20) (Santa Cruz; Cat. n.sc-6216) were used to ensure equal protein loading. HRP conjugated anti-rabbit (SIGMA; Cat. n.A 6154) and anti-mouse (ADVANSTA; Cat. n.R-05071-500) secondary antibodies were used, (1:5000 in 2% BSA) for 30 min. The chemiluminescent signal was detected using WesternBright^®^ ECL (ADVANSTA, San Jose, CA, USA; Cat. n.K-12045-D20). Densitometry analysis was performed with ImageJ Software (v. 10.2). Immunoblots were repeated three times with the same lysates and with protein lysates derived from three different treatments of olaparib and irradiation. Additional details of immunoblotting conditions can be found in Supplementary Materials and Methods.

### 2.8. Nuclear and Cytoplasmic Protein Extraction

Nuclear and cytoplasmic protein fractions were extracted from 3 × 10^6^ miR-200c-SKOV3 and pCMV-SKOV3 cells, untreated (DMSO) and treated with 1.5 μM or 5 μM olaparib or in combination with ionizing radiation (IR). The cells were washed in PBS twice and were pelleted at 1500 RPMI for 5 min. The cell pellets were lysed in 100 μL of Cytoplasmic Extract buffer (CE): with 10 mM HEPES, 60 mM KCl, 1 mM EDTA, 0.075% (*v*/*v*) NP-40, 1mM DTT, 1 mM PMSF, and adjusted to pH 7.6. The preparations were incubated on ice for 3 min, and then centrifuged at 1300 rpm for 5 min. The supernatants containing cytoplasmic extracts were collected in clean tubes, and re-centrifuged at a maximum speed for 10 min to pellet any remaining nuclei. The remaining nuclear pellets were washed with 100 μL of CE buffer without NP-40 detergent, suspending gently to maintain intact the fragile nuclei and centrifuged as above. After discarding the supernatant, nuclei were lysed in 50 μL of Nuclear Extract buffer (NE): 20 mM Tris Cl, 420 mM NaCl, 1.5 mM MgCl_2_, 0.2 mM EDTA, 1 mM PMSF and 25% (*v*/*v*) glycerol, and adjusted to pH 8.0. Salt concentration was adjusted in each tube to 400 mM using 35 μL of 5 M NaCl. Finally, glycerol at 20–25% (*v*/*v*) was added in the supernatants containing the cytoplasmic or nuclear protein fractions and the samples were stored at −80 °C. 

### 2.9. MiR-200c-3p and PD-L1, β-catenin, c-Myc Expression Correlation Studies in Cancer Cell Line Encyclopedia (CCLE)

Expression of miR-200c-3p and of *CD274*, *CTNNB1* and *MYC* genes, coding for PD-L1, β-catenin and c-Myc respectively, were analyzed in publicly available data from the most recent datasets of the Cancer Cell Line Encyclopedia (CCLE) portal [36]. These data were downloaded from CCLE data portal (https://portals.broadinstitute.org/ccle/data, accessed on 5 January 2021), specifically RNA-seq data normalized with the (RNA-Seq by Expectation-Maximization) RSEM algorithm [37] to quantify both gene and transcript expression levels (files CCLE_RNAseq_rsem_genes_tpm_20180929.txt, CCLE_RNAseq_rsem_transcripts_tpm_20180929.txt) and normalized expression on microRNA profiling from the Nanostring platform (file CCLE_miRNA_20181103.gct). Matched miRNA and mRNA expression sequencing data were retrieved across 46 different untreated OC cell lines, including SKOV3. Spearman correlation analysis was performed between hsa-miR-200c-3p and CD274, CTNNB1 and MYC genes and relative transcripts. The CCLE data portal also provides protein expression data from Reverse Phase Protein Arrays (RPPA, file CCLE_RPPA_20181003.csv), which were considered to check whether the miRNA effect could also be transcriptional. However, only β-catenin and c-Myc protein expression profiles are available on the CCLE data portal. Analyses and visualization were performed in R language (v. 3.5.1), using the R package ggstatsplot. For all statistical tests, *p*-values less than 0.05 were considered statistically significant.

### 2.10. Immunohistochemistry of EOC Paraffin Sections

PD-L1, c-Myc, β-catenin and CD3 protein expression was assessed by immunohistochemistry on formalin-fixed paraffin-embedded (FFPE) EOC tissue sections [38]. Protein expression was detected by exploiting Ventana primary antibodies: MYC (Y69 clone; Cat. n.790-4628), β-catenin (Cat. n.760-4242), PD-L1 (sp142 clone; Cat. n.740-4859) and CD3 (2GV6 clone; Cat. n.790-4341). All of the procedures were carried out automatically on Bench Mark Ultra (Ventana, Monza, Italy) using extended antigen retrieval and DAB as chromogen. The following tissues were used as positive controls: normal colon for MYC, breast for β-catenin and tonsil for PD-L1 and CD3. As negative control, non-immune mouse serum was used instead of the primary antibodies, as previously reported [38]. To quantify the expression and staining intensity of PD-L1, c-Myc and β-catenin, we performed ImageJ analysis of three different areas from each pre- and post-chemotherapy sample. The images were acquired at 20× magnification using a NanoZoomer digital slide scanner (Hamamatsu photonics Italia S.r.l., Arese, Milan). An unpaired *t* test was applied to demonstrate that differences in % total PD-L1, c-Myc and β-catenin-positive cells between the pre- and post-therapy biopsies were statistically significant.

### 2.11. Statistical and Bioinformatics Analyses

For RT-qPCR and immunostaining data with pairwise comparisons, a two-tailed unpaired *t*-test was applied to estimate *p*-values of the mean fold change for each gene expression, using Prism 7 software. For densitometry, clonogenic analysis and RT-qPCR data with multiple comparisons, one-way ANOVA followed by Dunnett’s test were applied, using Prism 7 software.

For bioinformatics analyses on cancer cell lines, RNA-seq data normalized with RNA-Seq by Expectation-Maximization (RSEM) algorithm [37] to quantify both gene and transcript expression levels (files CCLE_RNAseq_rsem_genes_tpm_20180929.txt and CCLE_RNAseq_rsem_transcripts_tpm_20180929.txt), normalized expression on microRNA profiling from Nanostring platform (file CCLE_miRNA_20181103.gct) and protein expression profiling from Reverse Phase Protein Arrays (RPPA, file CCLE_RPPA_20181003.csv) were considered from the CCLE data portal. For bioinformatics analyses on ovarian cancer tissues, RSEM-normalized RNA-seq and miRNA data normalized for batch-effects were downloaded from The Cancer Genome Atlas (TCGA), and the Pan-Cancer project (https://gdc.cancer.gov/about-data/publications/pancanatlas, accessed on 5 January 2021). Samples showing BRCA1 and/or BRCA2 non-silent somatic mutation were filtered out. T-cell infiltration, endothelial cells and cancer-associated fibroblast infiltration scores were estimated from the RNA-seq data using MCP-counter, a gene-expression-based tumor microenvironment deconvolution tool [39]. Spearman correlation analysis and scatter plots on CCLE and TCGA data were done in R language (v. 3.5.1), using the R package ggstatsplot (v. 0.6.5). For all statistical tests, *p*-values less than 0.05 were considered statistically significant.

## 3. Results

### 3.1. MiR-200c-3p and PDL1 Expression in EOC Biopsies before and after Chemotherapy 

**Figure 1:**
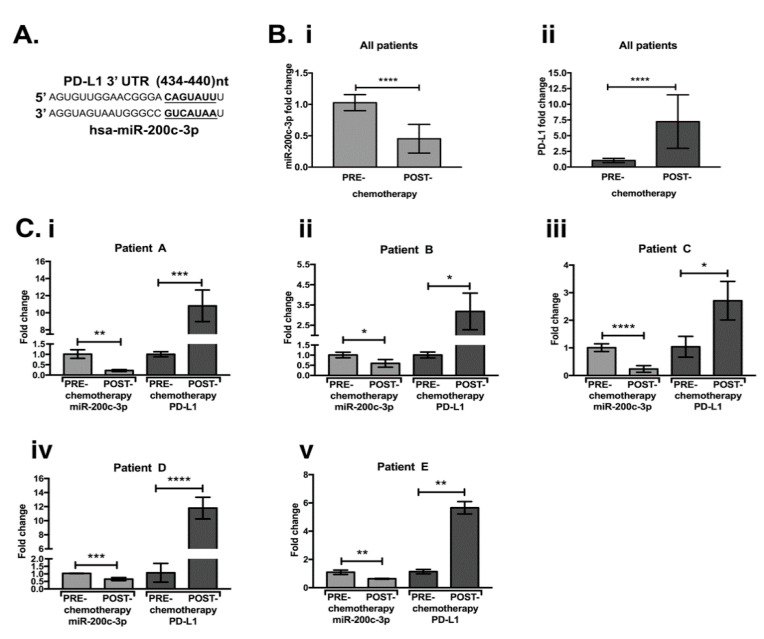
MiR-200c-3p and PD-L1 expression by RT-qPCR are inversely correlated in a cohort of OC patients’ biopsies. (**A**): TargetScan v7.1. prediction of miR-200c-3p seed sequence, highlighted in bold letters and underlined, complements the 3′UTR of PD-L1, from 434 to 440 nucleotides (nt). (**B**) (**i**): miR-200c-3p and (**ii**) PD-L1 expression in a pool of five OC laparoscopic biopsies before and after chemotherapy. (**C**) (**i**–**v**): miR-200c-3p and PD-L1 expression analysis in the OC biopsies, comparing pre-chemotherapy to post-chemotherapy treatment in each patient (A–E). Fold change was calculated as the average of miR-200c-3p and PD-L1 expression from all five OC patients. miR-200-3p and PD-L1 fold change expression, was normalized by the housekeeping genes RNU6 and GAPDH, respectively. Fold change of miR-200c and PD-L1 expression was calculated for each OC patient as 2^−ΔΔCt^, considering the difference of Ct (ΔCt) mean values for each gene in the pre-chemotherapy biopsies compared to the mean of the Ct values in the post-chemotherapy biopsies. RT-qPCR measurements were repeated at least three times and in technical triplicates. Two-tailed unpaired *t*-test was applied for statistical significance, * *p* < 0.05, ** *p* < 0.01, *** *p* < 0.001, **** *p* < 0.0001.

We first examined if PD-L1 is a predicted target of miR-200c-3p through the TargetScan v7.1 algorithm [40]. A miR-200c-3p binding site was found in the 3′UTR of PD-L1 transcript variant 1 (NM_014143.3), from 434 nt to 440 nt (Figure 1A). To assess the effect of chemotherapy on PD-L1 and miR-200c-3p expression, we performed RT-qPCR in a cohort of five EOC patients (Table 1) before and after chemotherapy (Figure 1). We noticed that the difference in miR-200c (Figure 1(Bi)), and PD-L1 (Figure 1(Bii)) levels was statistically significant, in the pool of EOC biopsies before and after the chemotherapy. The relative fold change of miR-200c-3p and PD-L1 for each patient after chemotherapy is shown in Figure 1(Ci–v). These results suggest that the decreased expression of miR-200c-3p might have most likely contributed to PD-L1 induction due to chemotherapy.

### 3.2. PD-L1 Is Targeted by miR-200c-3p in SKOV3 Cell Line

**Figure 2:**
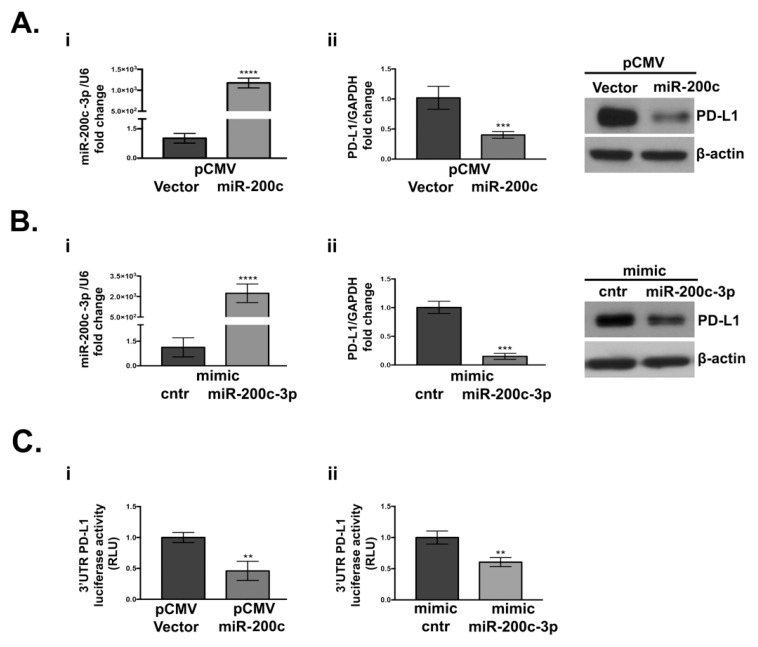
PD-L1 expression decreases upon miR-200c overexpression in SKOV3 cell line. (**A**) (**i**): RT-qPCR analysis of miR-200c-3p expression in miR-200c-stably transfected cells (pCMV-miR-200c). (**ii**): PD-L1 expression is reduced at both transcriptional and protein level, as indicated by RT-qPCR and Western blot (WB). (**B**) (**i**): miR-200c-3p expression in transiently transfected cells with mimic miR-200c-3p and mimic control (cntr), at 48 h, by RT-qPCR. (**ii**): At the same time point (48 h) PD-L1 transcript and PD-L1 protein are downregulated. MiR-200c-3p and PD-L1 fold change expression were normalized to the housekeeping genes RNU6 and GAPDH, respectively. The fold change analysis was performed, and expression values are reported as 2^−ΔΔCt^. β-actin was used as loading control. (**C**) (**i**): Decrease of the 3′UTR of PD-L1 luciferase activity, measured as relative luminometer units (RLU) of firefly-normalized to renilla in miR-200c transfected cells, relative to the empty vector-transfected cells. (**ii**): A decrease of PD-L1 3′UTR luciferase activity also occurs in the SKOV3 cell line transiently transfected with the mimic miR-200c-3p relatively to the mimic control (cntr). Luminescence detection was performed at 48 h with GloMax Luminometer (Promega). The experiments were repeated at least thrice and in six technical replicates. Two-tailed unpaired *t*-test was applied in each analysis for statistical significance, ** *p* < 0.01, *** *p* < 0.001, **** *p* < 0.0001.

To confirm that PD-L1 is an authentic miR-200c-3p target, we stably transfected SKOV3 with pCMV-miR-200c or the empty vector. The choice of SKOV3 cells was prompted by the fact that akin to EOC biopsies previously described, it expresses low miR-200c and moderate PD-L1, and it resembles advanced stage serous OC [41]. RT-QPCR analysis (Figure 2(Ai)) confirmed higher levels of miR-200c-3p in SKOV3-pCMV-miR-200c transfectants. A significant reduction of PD-L1 was noted in these cells at both transcriptional and protein levels (Figure 2(Aii)). To check if miR-200c-3p is responsible for PD-L1 down-regulation, SKOV3 cells were transiently transfected with a mimic miR-200c-3p. As seen in Figure 2(Bi,ii), at 48 h post-transfection, PD-L1 RNA and protein expression were decreased. Luciferase activity of psiCHECK™-2 vector 3′UTR PD-L1 reporters decreased in miR-200c and miR-200c-3p overexpressing cells in comparison to the vector transfected cells (Figure 2(Ci,ii)). These results confirm the interaction of miR-200c-3p with 3′UTR of PD-L1. Additionally, the higher miR-200c-3p expression did not have any further impact on PD-L1 at the protein level (Figure 2(Bii)), suggesting that the PD-L1 decrease is not miR-200c-3p dose dependent.

### 3.3. PD-L1, β-Catenin (CTNNB1) and c-Myc Expression and Its Correlation with miR-200c-3p in OC Cell Lines through CCLE Data Portal

**Figure 3:**
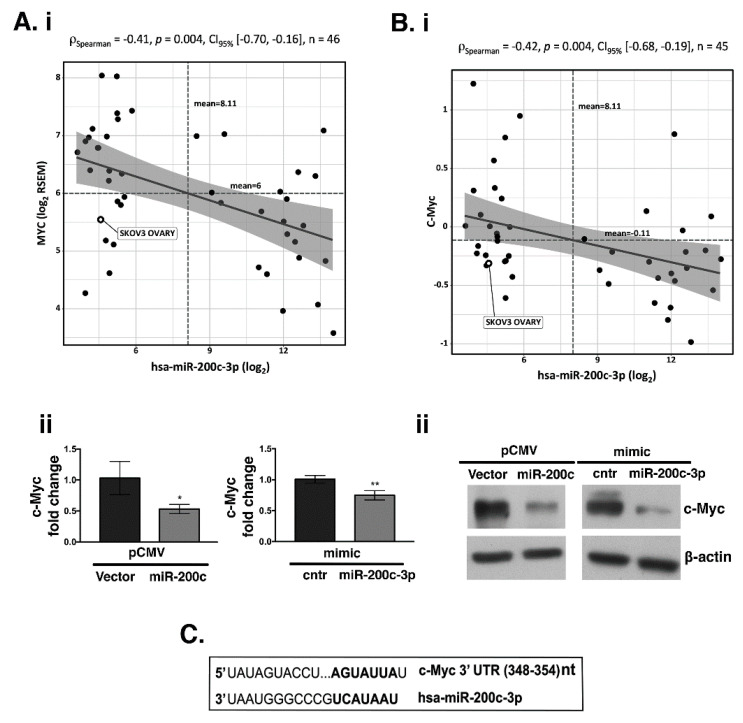
Transcriptional and translational regulation of c-Myc by miR-200c-3p (**A**) (**i**): RNA-sequencing data retrieved from CCLE data portal of 46 cell lines showed an inverse correlation between MYC gene and miR-200c-3p. (**ii**): RT-qPCR was used to verify the decrease of c-Myc transcript in both, stably transfected (pCMV V. and miR-200c) and transiently transfected (mimic cntr and mimic miR-200c-3p). SD± shows the mean of three independent experiments and in technical triplicates. * *p* < 0.05, ** *p* < 0.01, (**B**) (**i**): Reverse Phase Protein Arrays (RPPA) extracted from CCLE data portal in 45 OC cell lines as in A, showed that c-Myc protein is inversely correlated with miR-200c-3p. Spearman correlation analysis was reported, with statistical significance (*p* < 0.05). Confidence intervals at 95% are also reported. (**ii**): WB analysis of c-Myc expression in stably transfected (pCMV V. and miR-200c) and transiently transfected (mimic control: cntr and mimic miR-200c-3p). (**C**): TargetScan v7.1 prediction shows a miR-200c-3p binding site (seed sequence) in the 3′UTR of c-Myc from 358 to 354 nucleotides (top).

Previously published data showed that Wnt/β-catenin signaling is activated across different types of cancer and miR-200c down-regulates β-catenin expression in breast cancer [29]. Thus, we examined if there is an inverse correlation between miR-200c and β-catenin in EOC. A broad assessment was performed across the 46 OC untreated cell lines from the CCLE data portal [42]. We did not find a negative correlation between PD-L1 and miR-200c-3p (Figure S1). However, two out of fifteen splice variants of the CTNNB1 transcript were significantly inverse correlated with miR-200c-3p expression (Figure S2A). Additionally, we asked if c-Myc, a downstream target of β-catenin, could be regulated by miR-200c-3p. MYC proto-oncogene and all its five splice variant transcripts were significantly inverse correlated with miR-200c-3p expression (Figure S2B), as well as c-Myc at the protein level (*p* Spearman = −0.42, *p* = 0.004; Figure 3B). To confirm the in silico analyses, we assessed the expression of c-Myc, at the mRNA and protein level by RT-qPCR (Figure 3(Aii)) and WB (Figure 3(Bii)), respectively. Our results showed that the presence of miR-200c decreased c-Myc either at the transcriptional or protein level, corroborating the in silico analyses. In support of the inverse correlation observed between c-Myc and miR-200c-3p, TargetScan prediction showed that there is a seed sequence of miR-200c-3p complementary to the 3′UTR of c-Myc (Figure 3C). On the other hand, according to TargetScan, β-catenin is not among miR-200c-3p targets. Furthermore, to better evaluate the inverse correlation of PD-L1, c-Myc, and β-catenin with miR-200c-3p we used another OC cell line, UWB1.289+BRCA1 (Supplementary Materials and Methods, Figure S3), carrying wtBRCA1 [43]. First, we verified by RT-qPCR, that miR-200c-3p was highly expressed in UWB1.289+BRCA1 compared to the SKOV3 cell line (Figure S3A). Secondly, UWB1.289+BRCA1 was transiently transfected with anti-miR-200c-3p. After 48 h hours post-transfection with miR-200c-3p inhibitors, expressions of all three proteins were restored, as it was shown by WB analysis (Figure S3).

Taken together, our results show that miR-200c-3p inhibits the expression of PD-L1, c-Myc and β-catenin in the OC cell lines, SKOV3 and UWB1.289+BRCA1.

### 3.4. MiR-200c-3p Inhibits Its Target Genes in Olaparib/Ionizing Radiation (IR) Treated and Untreated SKOV3 Cells

To further investigate the effect of miR-200c on its target genes PD-L1, c-Myc and β-catenin, we treated SKOV3 pCMV-miR-200c transfectants with 1.5 μM and 5 μM olaparib, and with 4 Gy IR, with each treatment alone or in combination. DMSO-treated SKOV3 pCMV Vector cells were used as controls.

**Figure 4:**
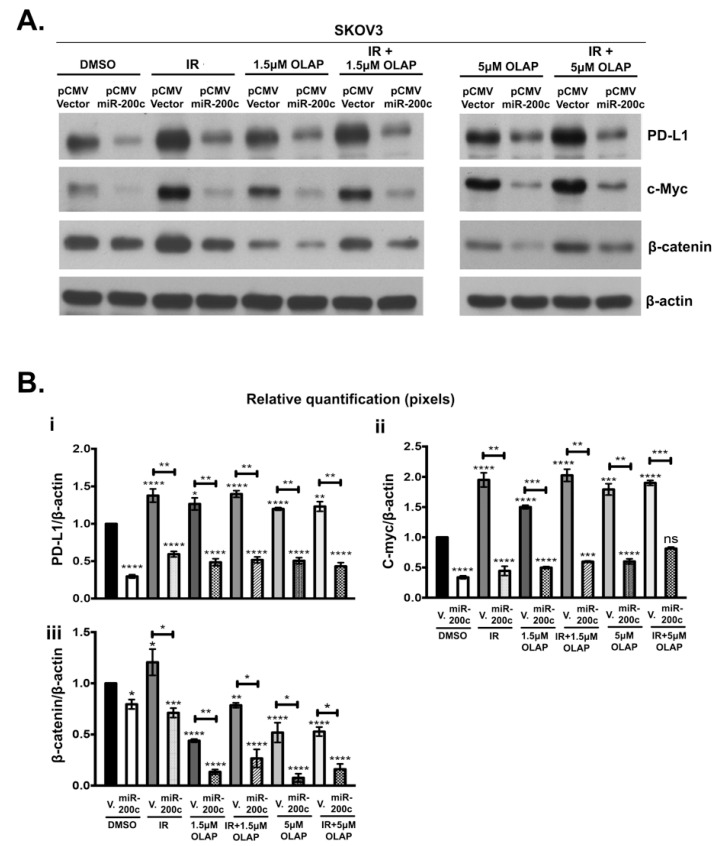
MiR-200c overexpression in SKOV3 transfected cells down-regulates PD-L1, c-Myc and β-catenin protein levels. (**A**): WB analysis of protein expression in SKOV3, transfected with pCMV-Vector and miR-200c and treated with DMSO, 1.5 μM or 5 μM of olaparib (OLAP), alone and in combination with 4 Gy irradiation (IR). β-actin is used as loading control. (**B**): (**i**–**iii**) Relative quantification of PD-L1, c-Myc and β-catenin by densitometry shows that when olaparib treatment is applied alone or in combination with IR, PD-L1 and c-Myc expression is induced only in the controls. β-catenin expression is induced only in pCMV Vector (V.) IR. The ratio of each protein/β-actin was used for normalization. MiR-200c decreases the expression of PD-L1, c-Myc and β-catenin, regardless of the type of treatment. WBs were repeated at least three times. *p*-values were calculated with Dunnett’s multiple comparison test, one-way ANOVA, compared to p CMV V. transfected cells treated with DMSO. Two-tailed unpaired *t*-test was applied between pCMV V. and the corresponding miR-200c cell line for statistical significance, * *p* < 0.05, ** *p* < 0.01, *** *p* < 0.001, **** *p* < 0.0001, ns: not significant.

Figure 4 shows WB analysis (A) and the corresponding densitometry (B) across different types of treatment. The presence of miR-200c downregulated PD-L1, c-Myc and β-catenin under all experimental conditions (Figure 4(A,Bi,Bii,Biii)). In the absence of miR-200c, IR alone increases expression of PD-L1, c-Myc and β-catenin (Figure 4A). Olaparib treatment at both concentrations induced a significant increase of c-Myc expression in pCMV Vector carrying SKOV3 cells (Figure 4(A,Bii)). In the same cell line treated with olaparib, PD-L1 expression was only marginally increased (less than 1.5 fold compared to the parental cell line), although this increase was statistically significant (Figure 4(A,Bi)). In contrast with PD-L1 and c-Myc expression, β-catenin expression was reduced by olaparib at either concentration. In contrast, IR treatment alone increased β-catenin. When both treatments were combined, the negative effect of olaparib on β-catenin expression was dominant. In all of the above treatments, the presence of miR-200c was able to counteract any increase of PD-L1, c-Myc and β-catenin expression.

### 3.5. Olaparib and IR Effect on miR-200c-3p and PD-L1 mRNA in SKOV3 Cells

**Figure 5:**
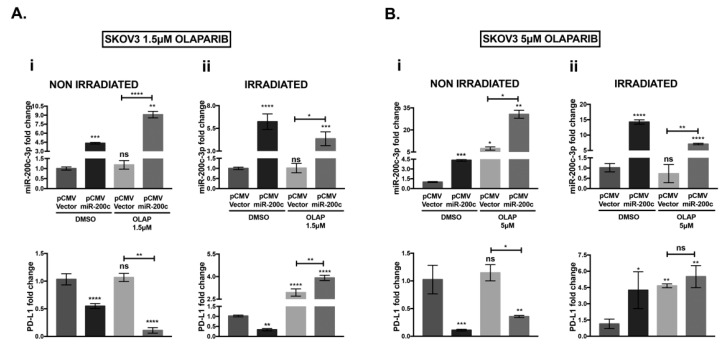
Analysis of miR-200c-3p and PD-L1 expression by RT-qPCR in SKOV3 cells treated with olaparib and irradiation. (**A**) (**i**): Non-irradiated: miR-200c-3p (upper-left graph) is inversely correlated with PD-L1 expression (bottom-left graph) in both DMSO and 1.5 μM olaparib-treated SKOV3 cells, (**A**) (**ii**): Irradiated: In DMSO-treated SKOV3 cells, miR-200c-3p and PD-L1 are inversely correlated. Co-treatment with 1.5 μM olaparib and irradiation shows an increase of PD-L1, regardless of high miR-200c expression. (**B**) (**i**): Non-irradiated: At the higher dose of olaparib, 5 μM, PD-L1 is inversely correlated to miR-200c-3p. (**B**) (**ii**): Irradiated: PD-L1 mRNA is induced in untreated and olaparib 5 μM-treated cells. Experiments were repeated three times and in technical triplicates. *p*-values were calculated by Dunett’s multiple comparisons: * *p* < 0.05, ** *p* < 0.01, *** *p* < 0.001, **** *p* < 0.0001, ns: not significant.

RT-QPCR analysis was performed to study if olaparib and IR treatments had any effect on miR-200c-3p and consequently on PD-L1 mRNA expression. The PD-L1 transcript was decreased when miR-200c-3p was over-expressed in non-irradiated SKOV3 cells, untreated (DMSO) and treated with 1.5 μM or 5 μM olaparib (Figure 5(Ai,Bi)), according to the results obtained by immunoblotting (Figure 4). At both doses, olaparib-treated SKOV3 miR-200c transfected cells significantly induced the levels of miR-200c-3p (forth bar) compared with the untreated counterpart (second bar) (shown in each graph of Figure 5(Ai,Bi)). Olaparib treatment at both concentrations seemed to have a marginal effect on mRNA levels of PD-L1 in SKOV3 pCMV V. (lower graphs, third bar in Figure 4(A,Bi)). Nevertheless, olaparib treatment induced PD-L1 protein expression in the same cell line, although this induction seemed marginal, it was statistically significant (Figure 4(A,Bi)). In SKOV3 miR-200c transfected cells, IR in combination with 1.5 μM and 5 μM olaparib, induced PD-L1 mRNA, followed by a small reduction of miR-200c-3p expression (forth bar) compared with the untreated SKOV3 miR-200c transfected cells (second bar) in Figure 5(Aii,Bii). This PD-L1 induction was only at the transcriptional level since, as previously shown in Figure 4(A,Bi), the levels of PD-L1 protein were significantly reduced by miR-200c-3p under the same conditions. In conclusion, even if IR induced the PD-L1 transcript, the presence of miR-200c-3p was able to maintain low PD-L1 expression.

### 3.6. MiR-200c Reduces Colony Formation Ability of SKOV3 Cells

**Figure 6:**
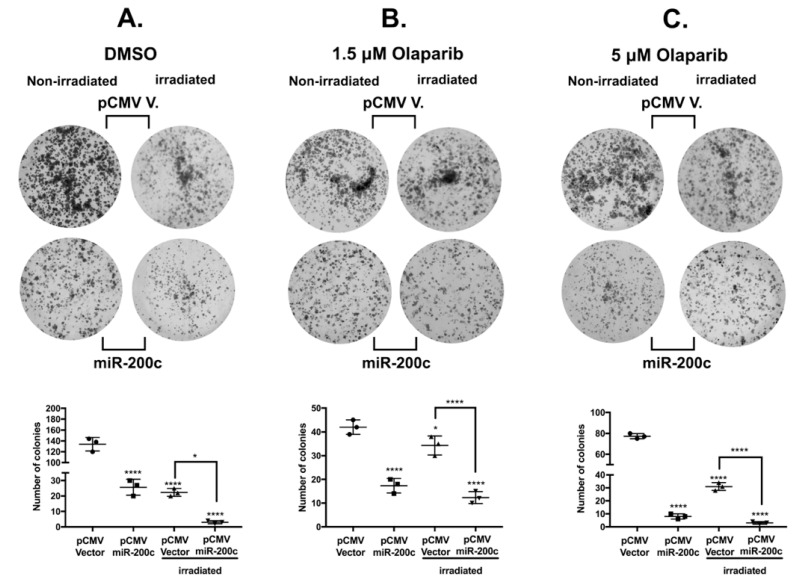
MiR-200c reduces colony formation of the SKOV3 cell line. (**A**–**C**): Representative images of one well out of three for each experimental condition of the colony formation assay, after two weeks. Under all of the experimental conditions: untreated (DMSO), irradiated only, 1.5 μM olaparib and 5 μM olaparib alone or in combination with irradiation, the number of colonies formation was reduced in the presence of miR-200c. The number of colonies in each experimental condition was determined by ImageJ, using the “analyze particles” command according to the software instructions. Particles considered as colonies under 100 mm^2^ pixels were excluded in order to measure only the largest colonies formed. The results of the scanned clonogenic assay images in A, B and C were plotted as means ± standard deviations of three separate experiments, each one with three replicates. Dunnett’s multiple comparisons test was applied for statistical significance in all the scatter dot plots. * *p* < 0.05, **** *p* < 0.0001.

In order to determine if miR-200c affects the clonogenic potential of SKOV3 cells treated with olaparib and IR in comparison with untreated cells, we performed a colony formation assay. We noticed that there was a cytotoxic effect of olaparib at both concentrations and IR alone (Figure 6A–C). Most importantly, the presence of miR-200c reduces the clonogenic ability of untreated and treated SKOV3 cells (second row in Figure 6A–C). Moreover, miR-200c reduced the colony formation significantly at the highest concentration of 5 μM olaparib, thus indicating that miR-200c sensitized SKOV3 cells to olaparib treatment, in accordance with previous results by our group [44].

We performed MTT assays at 24, 48 and 72 h to estimate the cell growth in a shorter period of time than the one used in the clonogenic assay (Figure S4A and Supplementary Materials and Methods). We noticed that the presence of miR-200c slows the proliferation of SKOV3 untreated cells, compared to the parental cell line. Since we showed, through MTT assay, that the difference in absorbance was significant at 72 h, we performed cell counting of SKOV3 pCMV V. and miR-200c transfected cells, seeded in a 6-well plate and in triplicates (0.3 × 10^6^ cells/well, for each condition). At 72 h, there was a significant reduction in the number of cells in the presence of miR-200c (Figure S4B). Furthermore, since miR-200c over-expression in SKOV3 cells decreased the expression of β-catenin and c-Myc, we assessed the expression of cyclin D1 (Figure S4C). Cyclin D1 is a downstream target of β-catenin and is involved in cell cycle progression in OC [45,46]. MiR-200c reduced the expression of cyclin D1. Our results corroborate the ones obtained through the clonogenic assays, that miR-200c slows proliferation of untreated and treated OC cells.

### 3.7. Subcellular Localization of β-Catenin and c-Myc Is Altered by miR-200c

**Figure 7:**
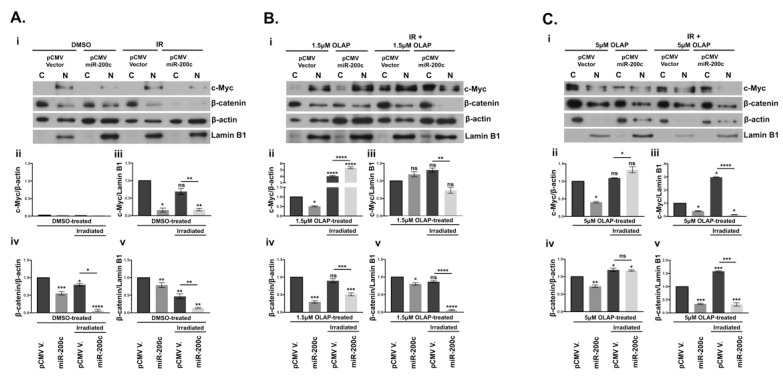
Effects of miR-200c on the distribution of c-Myc and β-catenin expressions in SKOV3 cell line. Cytoplasmic (C) and nuclear fractions (N) were analyzed by WB. β-actin and Lamin B1 are the housekeeping proteins used for cytoplasmic and nuclear extracts, respectively. (**A**) (**i**): empty vector (pCMV V.) and pCMV miR-200c SKOV3 cells treated with DMSO and 4 Gy irradiation (IR). (**B**) (**i**): vector and miR-200c transfected SKOV3 treated with 1.5 μM olaparib-only (OLAP) or in combination with 4 Gy irradiation. (**C**) (**i**): vector and miR-200c transfected SKOV3 treated with 5 μM olaparib-only (OLAP) treated vector and miR-200c or in combination with 4 Gy irradiation. The histograms reported below the WBs represent densitometric analysis by ImageJ of c-Myc and β-catenin, for each treatment. (**ii**,**iii**): the graphs indicate cytoplasmic and nuclear fractions of c-Myc respectively normalized to β-actin and Lamin B1 (**iv**,**v**): β-catenin expression in cytoplasmic and nuclear fractions. All the samples were normalized to the vector control SKOV3. The number of pixels from each protein signal imprinted on a film was normalized to the number of pixels of the respective housekeeping gene (β-actin or Lamin B1), calculated as a ratio. Three WB repetitions were performed with the same lysates and with protein lysates derived from three treatments upon olaparib and irradiation. Dunett’s multiple comparison statistical analysis was carried out with Prism 7 software, between pCMV V. DMSO/1.5μM OLAP/5 μM OLAP/IR-treated and the corresponding treated miR-200c transfected cells. * *p* < 0.05, ** *p* < 0.01, *** *p* < 0.001, **** *p* < 0.0001, ns: not significant.

As seen in Figure 4, protein expression of c-Myc and β-catenin was significantly down-regulated by miR-200c in SKOV3 cells. Since c-Myc is activated by β-catenin translocation from the membrane into the nucleus, we evaluated whether there was any differential expression of the nuclear and cytoplasmic fraction of these two proteins in miR-200c-SKOV3 cells [47]. Notably, our analysis of subcellular fraction proteins in Figure 7A–C demonstrated that while both c-Myc and β-catenin are expressed in the nucleus of the vector transfected cells, the presence of miR-200c significantly blocked their nuclear localization, upon combinatorial treatment with olaparib and irradiation (Figure 7(Aiii,Av,Biii,Bv,Ciii,Cv)). Olaparib treatment alone at both doses in the presence of miR-200c reduced expression of c-Myc and β-catenin in the nuclear fractions with one exception, at the lower dose of olaparib (Figure 7(Biii)). Interestingly, there was an accumulation of c-Myc in the cytoplasm when treated with olaparib and irradiation (Figure 7(Bii,Cii)). Similar cytoplasmic retention of β-catenin was seen in the presence of miR-200c in cells treated with the higher dose of olaparib (Figure 7(Civ)). Taken together, our data suggest that miR-200c could be instrumental in retention of both c-Myc and β-catenin in the cytoplasm.

### 3.8. Differential Expression of PD-L1, c-Myc, β-Catenin and CD3^+^ T-Cell Infiltration in Clinical Samples Obtained before and after Chemotherapy

**Figure 8:**
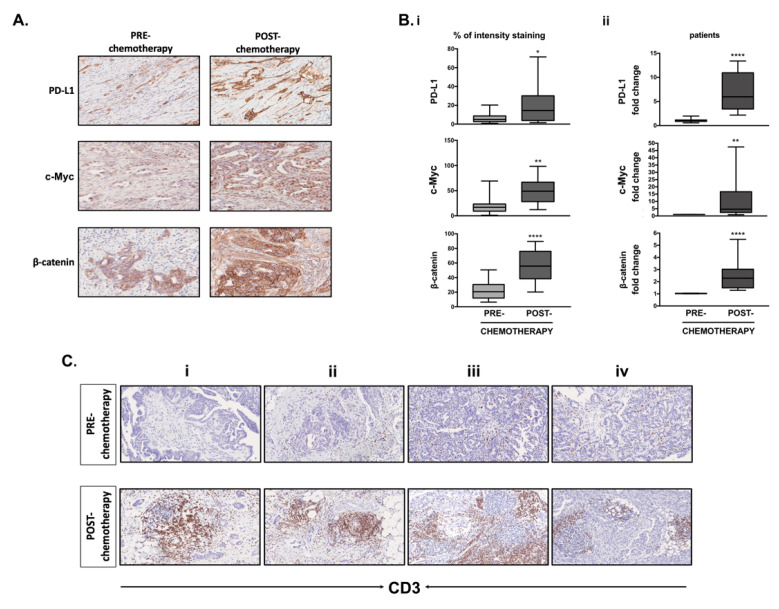
PD-L1, c-Myc, β-catenin expression and CD3+T cell infiltration increases in EOC patients’ biopsies after chemotherapy. (**A**) Formalin-Fixed Paraffin-Embedded (FFPE) tissue sections from all OC patients’ biopsies were immunoassayed for PD-L1, c-Myc and β-catenin staining using an automated Bench Mark Ultra (Ventana, Monza, Italy). One out of four representative images (Patient C) is shown. After extended antigen retrieval by heat-induced antigen retrieval (HIER), detection of the three proteins was performed with Ventana primary antibodies, using (3,3′-diaminobenzidine) (DAB) as chromogen. Deposition of brown staining reveals the presence of each protein. The stained tissue sections were digitalized at a 20× magnification using a NanoZoomer Digital Slide Scanner. (**B**) (**i**): The percentage of intensity staining for each protein was evaluated by counting the total PD-L1, β-catenin and c-Myc positive cells (neoplastic and stromal cells) in three squared areas from each clinical sample, using ImageJ. The % of immunostaining intensity from four biopsies pre- and post-chemotherapy was graphically calculated as a mean of four samples. Two tailed unpaired t test demonstrated the statistically significant differences among pre- and post-chemotherapy biopsies, * *p* < 0.05, ** *p* < 0.01, **** *p* < 0.0001. (**ii**): RT-qPCR analysis of PD-L1, c-Myc and β-catenin transcripts was done by pooling together all the biological replicates from the five patients. Each graph represents the mean standard deviation of c-Myc and β-catenin fold change, normalized to GAPDH housekeeping gene, from three independent experiments and in technical triplicates. Statistical significance was calculated using two tailed unpaired *t* test through PRISM7. **** *p* < 0.0001, ** *p* < 0.01 (**C**). CD3^+^ T cell infiltration in the tumor area of each biopsy, in four patients (**i**–**iv**) pre- and post-chemotherapy. The brown staining reveals an increase of CD3^+^ T cell infiltration in post-chemotherapy samples. A marked increase of peritumoral CD3^+^ T-cells, sometimes organized into follicular structures, was reported in post-chemotherapy biopsies in respect to pre-chemotherapy specimens.

Since miR-200c-3p was inversely correlated with PD-L1 expression before and after chemotherapy, we assessed PD-L1, c-Myc and β-catenin expression by IHC and RT-qPCR in the same biopsies (Figure 8(A,Bi,Bii)). Chemotherapy increased PD-L1 expression in all of the neoplastic cells with complete membrane reinforcement compared to a weak cytoplasmic positivity with partial membrane reinforcement in 10% of the neoplastic cells before chemotherapy (Figure 8(A,Bi), first panel). c-Myc nuclear positivity was increased in 70–80% of neoplastic cells in post-chemotherapy biopsies compared to a weak nuclear positivity in 10–30% of neoplastic cells before chemotherapy (Figure 8(A,Bi), second panel). β-catenin showed a complete membrane staining in all neoplastic cells after chemotherapy (Figure 8(A,Bi), third panel). The overall increase of c-Myc and β-catenin in the post-chemotherapy patient’s biopsies was further confirmed by RT-qPCR (Figure 8(Bii), pooled graphs of the five patients and Figure S5, graphs for each patient). Figure 8(Bii) shows the same graph of PD-L1 expression in all five patients pooled together, which was previously shown in Figure 1(Bii).

In EOC patients, tumor infiltrated lymphocytes (TILs) might be associated with improved overall survival [48], but at the same time stromal cells of the tumor microenvironment (TME) might generate an immunosuppressive state [49]. Figure 8(Ci–iv) shows that after chemotherapy, peritumoral CD3^+^ T cell infiltration increases, sometimes organized into follicular structures. To assess if there is any correlation between PD-L1, c-Myc, and β-catenin expression with T-cell infiltration we performed correlation analysis by using the Microenvironment Cell Populations-counter method of TCGA datasets of EOC patients (Supplementary Materials and Methods and Figure S6) [39]. We found a trend of positive correlation between PD-L1 expression and T-cell infiltrates, whilst MYC and β-catenin were inverse correlated with T-cell infiltrates (Figure S6(Ai–iii)). Using the same dataset, it was noted that a higher miR-200c-3p expression correlates with less abundant tumor-associated endothelial cells and cancer-associated fibroblasts (CAFs) (Figure S6(Bi,ii)). Taken together, the IHC and TCGA data suggest that an increased PD-L1, MYC and β-catenin expression might favor a less immunogenic TME and low levels of miR-200c-3p might characterize a more aggressive TME in EOC patients [49].

## 4. Discussion

MiR-200c is one of the most deregulated and well-studied miRNAs in OC. However, there is disagreement as to whether it is a tumor suppressor miRNA since its overexpression was correlated with shorter overall survival in EOC patients [50]. Therefore, it becomes imperative to clarify the role of miR-200c in the present context. Our data contribute the following critical insights to the ongoing debate. We identified an inverse correlation between miR-200c-3p, PD-L1 and two oncogenes, c-Myc and β-catenin, in EOC patients who had undergone chemotherapy with carboplatin/paclitaxel. We confirmed that c-Myc and β-catenin were significantly inverse correlated with miR-200c-3p, analyzing in silico data from the Cancer Cell Line Encyclopedia (CCLE), in 46 ovarian cancer cell lines. Notably, from the same database, we found a significant inverse correlation between c-Myc and miR-200c-3p, both at transcriptional and translational level, and it was confirmed in SKOV3 stably transfected with miR-200c. This result was also supported by TargetScan prediction. Most significantly, we found that miR-200c overexpression in the SKOV3 cell line inhibited PD-L1, c-Myc and β-catenin expression. We further validated these results in another EOC cell line, UWB1.289+BRCA1, which exhibits high endogenous levels of miR-200c-3p and moderate levels of PD-L1. In this cell line, inhibition of miR-200c-3p de-repressed the expression of PD-L1, c-Myc and β-catenin. Subsequently, we investigated the reason behind the induction of PD-L1, c-Myc and β-catenin expression observed in clinical samples derived from patients who underwent chemotherapy. Several studies support the same induction of PD-L1 by not only chemotherapy, but by olaparib and irradiation therapies as well [51,52,53]. Hence, we treated SKOV3 cells with olaparib, radiation, and both combined, to investigate any increase of PD-L1, c-Myc and β-catenin expression, which might worsen the outcome of these therapies. We provided experimental evidence that miR-200c slows proliferation of untreated and treated OC cells, by inhibiting PD-L1, c-Myc and β-catenin expression.

In olaparib-treated parental SKOV3 cells, the expression of PD-L1 was significantly induced, although to a lesser extent compared to c-Myc expression. Our data are in line with a previous study that showed PD-L1 induction mediated by PARPi in breast cancer cell lines and in mice models and rendered PARPi-treated breast cancer cells resistant to T-cell killing [54]. Another study showed that WNT/β-catenin activation in EOC cell lines contributed to PARPi resistance, but SKOV3 was not among these cell lines [55]. In contrast, we showed that, the SKOV3 cell line had increased basal levels of β-catenin and after olaparib treatment β-catenin expression was significantly decreased. This might explain the reduced clonogenic potential of olaparib-treated SKOV3 cells. As seen for PD-L1, IR and olaparib treatment significantly increased c-Myc protein levels. In spite of this increase in c-Myc, the treated SKOV3 cells showed less colony formation. This is most likely due to the fact that deregulated c-Myc expression induces apoptosis as a safeguard towards unwarranted cellular proliferation. Our observation of low clonogenicity in spite of higher c-Myc is consistent with pro-apoptotic functions of c-Myc [56]. The presence of miR-200c in either treated or untreated SKOV3 cells, was sufficient to reduce their clonogenicity and proliferation and further sensitized them to olaparib and IR treatment [44]. Based on our results, we suggest that miR-200c-3p might decrease c-Myc expression directly by targeting the 3′UTR. Interestingly, c-Myc represses the miR-200 family by directly binding to its promoter, which has been confirmed in endometrial carcinoma [57]. Our data showed that miR-200c-3p can negatively regulate c-Myc, suggesting a reciprocal regulation through a negative feedback loop between c-Myc and miR-200c-3p. Interestingly, Casey et al. have recently shown that c-Myc induces PD-L1 expression [58]. On the other hand, a recent study showed that PD-L1 positively regulates β-catenin expression and EMT, suggesting a pro-tumorigenic role of PD-L1 in lung cancer [59]. We cannot exclude the possibility that PD-L1 might have a similar function in OC. In addition, accumulating evidence shows that the Wnt/β-catenin pathway suppresses the immune response within the tumor microenvironment [60]. Based on these studies, it is tempting to speculate that c-Myc might induce PD-L1, which in turn induces β-catenin, enhancing tumor progression and immune-evasion. MiR-200c-3p might act as a double-edged sword by reducing PD-L1 directly by binding to its 3′UTR and indirectly through downregulation of c-Myc. At the same time, miR-200c-3p can target β-catenin directly, or through targeting PD-L1. Hence, the results in the present study corroborate miR-200c-3p as a tumor suppressor in EOC.

In the primary tumors, chemotherapy restricted the tumor area into a dense fibrotic stroma but did not completely eliminate it, thus indicating a good response to the treatment as also reported previously [61]. However, there was a significant increase in the percentage (%) of PD-L1, c-Myc and β-catenin positive cells (tumor cells and stromal cells) after chemotherapy treatment. Induction of β-catenin in EOC is a marker of poor prognosis [62]. While constitutive expression of c-Myc protein has become a clinical biomarker for the diagnosis and prognosis of aggressive B-cell lymphoma [63], most likely in advanced EOC patients, it could be an independent prognostic factor. However, a study showed that c-Myc protein levels were higher in cisplatin-resistant cells and may be a potential therapeutic target of OC [64]. Similarly, the possibility that the increased expression of c-Myc observed in the biopsies after chemotherapy and in vitro treated SKOV3 cells could be due to the presence of OC cells resistant to all of these treatments can certainly not be excluded.

An increase in PD-L1 expression accompanied by a strong increase of CD3^+^ T cell infiltration after chemotherapy suggested that the treatment is most likely responsible for the increase in number of TILs in the surrounding microenvironment. The presence of TILs and high PD-L1 expression in the TME might be an indicator of impaired function of CD4^+^ and CD8^+^ T cells against tumor cells [48,65] or might be associated with improved OS and PFS in EOC patients [66,67]. Since Wnt/β-catenin signaling is involved in immune evasion in OC as PD-L1 is, the increase of β-catenin along with PD-L1 in the biopsies of chemotherapy-treated patients, might worsen the outcome of these therapies. Notwithstanding the small cohort and lack of treatment comparison in the TCGA analysis, our data lend support to the notion that a decreased miR-200c-3p and the concomitant increase in PD-L1, c-Myc and β-catenin after chemotherapy could lead to an immunosuppressive TME and might be an indicator of worse prognosis. Furthermore, our data provide evidence that reconstitution of miR-200c-3p tumor suppressor function in EOC might contrast with the induction of PD-L1, c-Myc and β-catenin expression by carboplatin/paclitaxel, olaparib and radiation treatments.

In conclusion, since miRNA-based clinical trials are gaining traction [19], miR-200c-based therapy, either alone or in combination with conventional therapeutic regimens, might provide additional means to reconstitute anti-tumor immunity and contrast a pro-tumor microenvironment and progression.

## Figures and Tables

**Table 1:** Overview of HGSOC patients. International Federation of Gynecology and Obstetrics (FIGO) classification.

Patient	Age	Grade	FIGO	Chemotherapy Regimen	BRCA1/2 Status
A	56	G3	IV	Carboplatin auc 5 ^a^ + TXL 175 ^b^ + BEVA 15 ^c^ (3 cycles)	WT
B	43	G3	IIIC	Carboplatin auc 5 + Caelyx 30 ^d^ (3 cycles)	mutBRCA1 class 5
C	46	G3	IIIC	Carboplatin auc 5 + TXL 80 (3 cycles)	WT
D	69	G3	IIIC	Carboplatin auc 5 + TXL 175 (3 cycles)	WT
E	55	n.d.	IIIC	Carboplatin auc 3 + TXL 80 (cycles n.d.)	WT

Grade 3: (high grade) poorly differentiated ovarian epithelial cancer cells. FIGO classification: IIIC: Tumor involves one or both ovaries with macroscopic (>2 cm) cytologically or histologically confirmed metastasis to the peritoneum outside the pelvis and/or to the retroperitoneal lymph nodes. IV: Distant metastasis excluding peritoneal metastasis. Includes hepatic parenchymal metastasis. ^a^ AUC: area under the curve, ^b^ TXL: paclitaxel 175 mg/m^2^, ^c^ BEVA: bevacizumab, ^d^ Caelyx: liposomal doxorubicin 30 mg/m^2^, WT: wild type, mutBRCA1: mutatedBRCA1.

## Data Availability

Data is contained within the article or supplementary material. The data presented in this study are available in supplementary material at www.mdpi.com/xxx/s1.

## Supplementary Materials

The following are available online at https://www.mdpi.com/2073-4409/10/3/519/s1, Figure S1: Correlation between CD274, gene coding for PD-L1, and miR-200c-3p expression across the Ovarian Cancer Cell Lines from CCLE database. Figure S2: Inverse correlation of β-catenin (A) and c-Myc (B) transcripts with miR-200c-3p expression across the Ovarian Cancer Cell Lines from CCLE database. Figure S3: Inhibition of miR-200c-3p in UWB1.289+BRCA1 cell line restores expression of PD-L1, c-Myc and β-catenin. Figure S4: MiR-200c inhibits proliferation of SKOV3 cells. Figure S5: Overall increased expression of c-Myc and β-catenin in OC patients after chemotherapy. Figure S6: TCGA analyses using MCP-counter of T-cell infiltration, endothelial cells and cancer-associated fibroblast infiltration score in EOC (HGSOC) patients, untreated and BRCA1/2 WT. Table S1: Description of 46 OC cell lines from Cancer Cell Line Encyclopedia (CCLE) used for the in silico analyses. Supplementary Materials and Methods.
