# Supplementary material for: MiR-200c-3p Contrasts PD-L1 Induction by Combinatorial Therapies and Slows Proliferation of Epithelial Ovarian Cancer through Downregulation of β-Catenin and c-Myc
Source: Cells. 2021 Mar 1;10(3):519. doi: 10.3390/cells10030519 (PMC7998372; doi:10.3390/cells10030519)
Supplement: Supplementary file 1 [file cells-10-00519-s001.zip › cells-1099740 supplementary proofreading back/Supplementary material/Supplementary figures and legends.docx]

**Supplementary Figures**


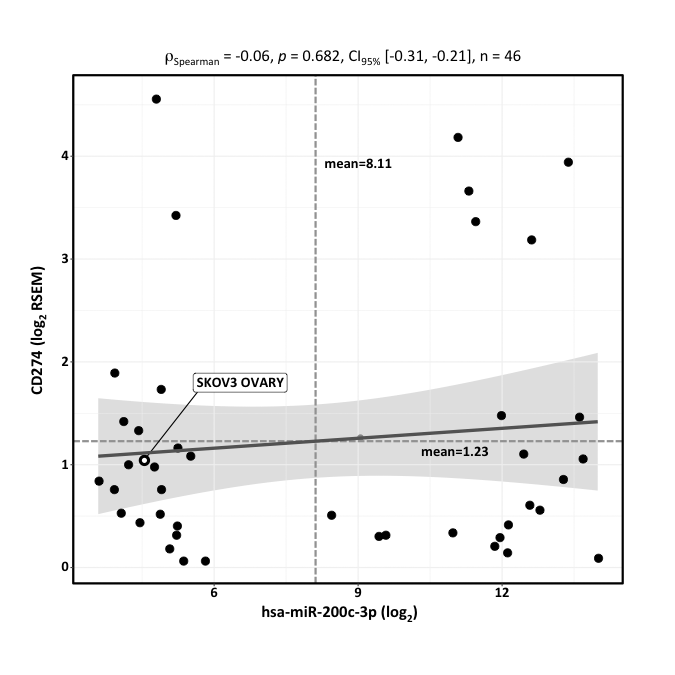


**Figure S1.** Correlation between CD274, gene coding for PD-L1, and miR-200c-3p expression across the Ovarian Cancer Cell Lines from CCLE database. No significant inverse correlation was observed (*p*-value=0.682). RSEM log2 expression of PD-L1 and log2 miR-200c-3p expression among all the OC cell lines were between 0.05 and 4.56 for PD-L1, and approximately 0.05 and 15 for miR-200c-3p expression. RSEM: RNA-Seq by Expectation-Maximization.





**Figure S2.** Inverse correlation of β-catenin (**A**) and c-Myc (**B**) transcripts with miR-200c-3p expression across the Ovarian Cancer Cell Lines from CCLE database. (**A**): Only two of the 15 β-catenin splice variants encoded in the human reference genome 19, indicated with Latin numbers viii and xii, were found to be anti-correlated with miR-200c-3p expression. The location of β-catenin gene is indicated with a light gray color sign in chromosome 3. (**B**): All five c-Myc transcripts, from i to v, identified in human reference genome 19, resulted inversely correlated with miR-200c-3p expression. The location of c-Myc gene is indicated with a light gray color sign chromosome 8. Matched miR-200c-3p and c-Myc mRNA expression sequencing data were retrieved across *n*=46 different untreated ovarian cancer cell lines, including SKOV3. Each grey point in the graphs represent a different OC cell line obtained from CCLE data portal (full list reported in Table S1). Spearman’s correlation coefficients (ρ) values indicate the inverse correlation between every transcript variant and miR-200c-3p, statistically significant for all the comparisons reported (*p*-values<0.05). Confidence intervals at 95% are also reported.


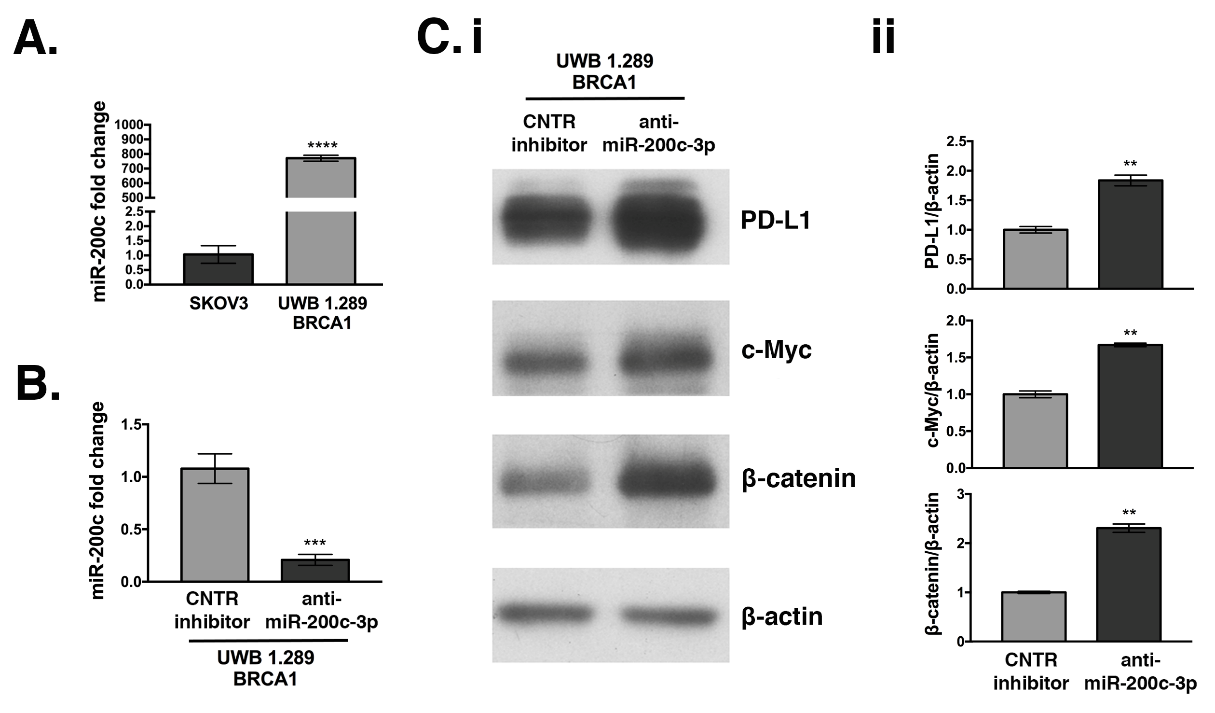


**Figure S3.** Inhibition of miR-200c-3p in UWB1.289+BRCA1 cell line restores expression of PD-L1, c-Myc and β-catenin. (**A**). RT-qPCR detection of miR-200c-3p in SKOV3 in comparison with UWB1.289+BRCA1 cell line. (**B**). Detection of miR-200c-3p by RT-qPCR in UWB 1.289+BRCA1 cell line transfected with anti-miR-200c-3p, to verify the K.D. Each graph represents the mean standard deviation of miR-200c-3p normalized to U6 housekeeping gene, from three independent experiments and in technical triplicates. Statistical significance was calculated using two tailed unpaired t test through PRISM7. **p<0.01, ***p<0.001, ****p<0.0001. (**C**). (i): Expression of PD-L1, c-Myc and β-catenin, by WBs analysis. (ii): Densitometry analysis was calculated as a ratio of each protein’s band normalized to the one of β-actin. Statistical significance was calculated using two tailed unpaired t test through PRISM7., ***p*<0.01.


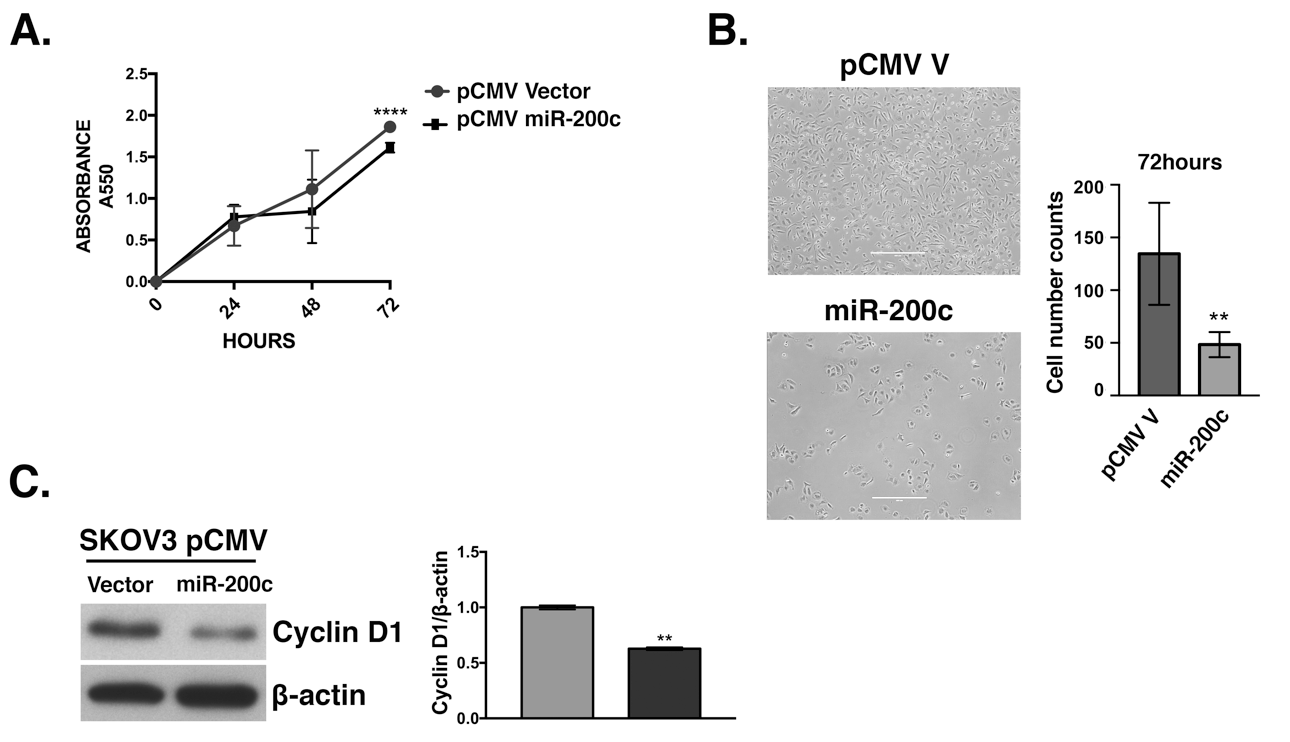


**Figure S4**. MiR-200c inhibits proliferation of SKOV3 cells. (**A**). MTT assay was performed at 24, 48 and 72h. SKOV3 pCMV V. and pCMV miR-200c transfected cells were seeded at 7 × 10^3^ cells/well in 96 well plate and in six replicates for each condition. Absorbance was measured at 550 nm, with reference at 630 nm, using a microtiter plate reader (Select Science). SD± is the mean of A550 of 6 replicates, for each condition. *****p* < 0.0001. (**B**). pCMV Vector and miR-200c transfected SKOV3 cells were seeded at 0.3 × 10^6^ each in triplicates, in a 6-well plate. The absolute cell number was counted at 72hours, to estimate differences in the cell number and phenotype. Representative photos for each condition were taken with EVOS XL Core Imaging System (ThermoFisher Scientific), using 10× magnification (white line scale is 400μm). SD ± is the mean of the cell number counts in three equal areas for each well and for each condition. ***p* < 0.01. (**C**). WB detection of Cyclin D1 in SKOV3 cells transfected with the Vector control (pCMV) and miR-200c carrying vector. β-actin was used as a loading control. One out of two WBs is shown. Densitometry analysis was performed with Image J and calculated as a Cyclin D1/β-actin ratio.





**Figure S5.** Overall increased expression of c-myc and β-catenin in OC patients after chemotherapy.(**i-v**): RT-qPCR analysis of c-myc and β-catenin transcripts in all five patients indicated as patient A to patient E. In every graph the fold change expression of c-myc or β-catenin after chemotherapy (post-) is compared to the corresponding gene expression before chemotherapy (pre-). Each graph represents the mean standard deviation of c-myc and β-catenin fold change, normalized to GAPDH housekeeping gene, from three independent experiments and in technical triplicates. Statistical significance was calculated using two tailed unpaired t test through PRISM7. ****p* < 0.001, ***p* < 0.01, **p* < 0.05, ns = not significant.





**Figure S6.** TCGA analyses using MCP-counter of T-cell infiltration, endothelial cells and cancer-associated fibroblast infiltration score in EOC (HGSOC) patients, untreated and BRCA1/2 WT. (**A**): Correlation studies between T-cell infiltration score and CD274 (PD-L1), A(i), MYC, A(ii) and CTNNB1 A(iii) across a number of *n* = 290 OC patients, BRCA1/2 WT. (**B**): Correlation studies of Endothelial cell infiltration (i) and Cancer-associated fibroblasts (ii) with the expression of miR-200c-3p across a number of *n* = 286 OC patients, BRAC1/2 WT. Spearman's correlation coefficients with related *p*-values and scatter-plots were generated using ggstatsplot R package (v. 0.6.5). *p*-values less than 0.05 were considered statistically significant.
