# Supplementary material for: MiR-200c-3p Contrasts PD-L1 Induction by Combinatorial Therapies and Slows Proliferation of Epithelial Ovarian Cancer through Downregulation of β-Catenin and c-Myc
Source: Cells. 2021 Mar 1;10(3):519. doi: 10.3390/cells10030519 (PMC7998372; doi:10.3390/cells10030519)
Supplement: Supplementary file 1 [file cells-10-00519-s001.zip › cells-1099740 supplementary proofreading back/Supplementary material/Supplementary materials and methods.docx]

**Supplemental materials and methods**

**Cell line**

The Human ovarian cancer cell line UWB1.289+BRCA1 is a papillary serous ovarian cancer, which is derived by stably transfection of wild-type BRCA1 in UWB1.289 null-BRCA1 cell line[1]. UWB1.289+BRCA1was purchased from ATCC® (CRL-2946™). This cell line was chosen for two reasons: 1) It owns high endogenous levels of miR-200c, and 2) has a restored BRCA1 wild-type (wt), which coincides with the status of

wt BRCA1 in SKOV3 cell line and that of the patients. We used this cell line to perform miR-200c-3p K.D. experiments.

**RT-qPCR**

Detection of PDL1 and GAPDH mRNA was performed with QIAGEN QuantiTect Primer Assay predesigned primers (Hs_CD274_1_SG QuantiTect Primer Assay, Cat. n. QT00082775; Hs_GAPDH_1_SG QuantiTect Primer Assay, Cat. n. QT00079247). Mature miR-200c-3p and U6 expression levels were detected using miScript Primer Assay (QIAGEN; Hs_miR-200c_1 miScript Primer Assay, Cat. n. MS00003752; Hs_RNU6-2_11, Cat. n. MS00033740). C-myc (primer ID: H_MYC_1) and β-catenin (primer ID: H_CTNNB1_1) amplification was performed with KiCqStart™ predesigned for SYBR green I primers (Sigma-Aldrich).

**Transfections**

UWB1.289+BRCA1 0.8x10^6^ cells were transiently transfected in a 6 well plate and in triplicates with 40 nM anti-miR-200c-3p oligonucleotide (MISSION®, Synthetic microRNA Inhibitor, Human, Sigma-Aldrich; Cat. n. HSTUD0354,) and the same amount of negative control based upon an Arabidopsis thaliana sequence (MISSION®, Sigma-Aldrich; Cat. n. NCSTUD001). DharmaFECT Duo Transfection Reagent (Dharmacon; Cat. n. T-2010-02) was used according to the manufacturer’s instructions. After 48 h, the cells were collected for RNA and protein extraction.

**Colony formation assay**

Clonogenic assays were repeated at least three times. The growth medium was removed, and adherent cells were washed twice with 2 ml of PBS. Subsequently, the cells were fixed by adding 1 ml of methanol (MERCK; Cat. n. 34860) per well, and the plates were left for 10 minutes at -20°C. After methanol removal, fixed cells were washed twice with PBS. Colonies were stained with 0.1 % crystal violet for 5 minutes at room temperature (RT) and after three washings with deionized water. Plates were left to dry overnight and the next day they were scanned. Images were elaborated with ImageJ software, using the “measure particles” command, according to the software instructions. The results were plotted as means of standard deviations of three separate experiments and in triplicates per assay for each experimental condition. Dunnett's multiple comparisons test was applied for statistical significance.

**MTT assay and cell counting**

The viability of SKOV3 pCMV Vector and pCMV miR-200c transfected cells was measured using the 3-[4,5-dimethylthiazol-2-yl]-2,5 diphenyl tetrazoliumbromide (MTT) assay. Cells were seeded at the density of 7×10^3^ cells/well in six replicates for each condition in a 96 well plate. At 24h, 48h and 72h, 0.5 mg/ml of MTT solution (Sigma-Aldrich; Cat. n. 11465007001) was added to each well and cells were incubated for 4h at 37 °C. Then, supernatant was discarded and in order to dissolve formazan crystals 100 μl of DMSO were added to each well. The plates were incubated at 37 °C for 10 minutes. Subsequently, the absorbance was measured at 550 nm, with reference at 630 nm, using a microtiter plate reader (Select Science). The results were plotted as a mean ± standard deviation of six replicates for each condition.

For a direct counting of living cells, 0.3x10^6^ pCMV Vector and pCMV miR-200c SKOV3 cells were seeded in triplicates in a 6 well plate. After 72h, 10X magnification pictures of three different fields for each condition were taken with EVOS XL Core Imaging System (ThermoFisher Scientific) and the absolute cell number was determine using ImageJ software [2]. Results were expressed as the mean of at least two different experiments.

**Immunoblotting**

Blotting was performed onto Immobilon-P PVDF membranes (Millipore; Cat. n. IPVH00010) at 20 V overnight at 4°C. Membranes were blocked with 5 % non-fat dried milk (Biorad; Cat. n. 1706404) and incubated overnight at 4 °C with the appropriate primary antibody dilution, in 5% skimmed milk. The number of pixels from each protein signal imprinted on a film was normalized to the number of pixels of the respective housekeeping gene (β-actin or Lamin B1), calculated as a ratio. Cyclin D1 (DCS-6) antibody (Santa Cruz; Cat. n sc-20044) was used. Statistical analyses of densitometry for Cyclin D1/β-actin ratios were calculated with Prism 7 software, using two-tailed unpair t-test.

**Correlation studies of T-cell infiltration, epithelial cells and Cancer-associated fibroblasts (CAFs) infiltration score in EOC patients retrieved from TCGA**

Expression of genes *CD274, CTNNB1 and MYC* (coding for PD-L1, β-catenin and c-Myc, respectively) in 290 and miR-200c-3p in 286 patients with High-Grade Serous Ovarian Carcinoma were retrieved from The Cancer Genome Atlas (TCGA). Specifically, sample-level normalized, log2 (normalized value +1)-transformed gene and miRNA expression data, adjusted for batch effects, were download from Sage Bionetworks’ Synapse Pan-cancer Atlas data browser. Samples of untreated patients were available in TCGA and analyzed, filtering out those who carried mutations either for BRCA1 or BRCA2, to be consistent with the cohort of patients and the SKOV3 cell line used in our studies. Microenvironment cell populations-counter (MCP-counter), an RNA-seq-based method which allows the quantification of immune and stromal cell populations in heterogeneous tissues[3], was performed for estimation of the association between the genes *CD274, CTNNB1, MYC*, miR-200c-3p and tumor-infiltrating immune/stromal cell populations, focusing on T-cell and endothelial and cancer-associated fibroblasts (CAFs) infiltration. Spearman's correlation coefficients with related p-values and scatter-plots were generated using ggstatsplot R package (v. 0.6.5). P-values less than 0.05 were considered statistically significant.

**References**

1. DelloRusso, C.; Welcsh, P.L.; Wang, W.; Garcia, R.L.; King, M.C.; Swisher, E.M. Functional characterization of a novel BRCA1-null ovarian cancer cell line in response to ionizing radiation. *Mol Cancer Res* **2007**, *5*, 35-45, doi:10.1158/1541-7786.Mcr-06-0234.

2. Schneider, C.A.; Rasband, W.S.; Eliceiri, K.W. NIH Image to ImageJ: 25 years of image analysis. *Nat Methods* **2012**, *9*, 671-675, doi:10.1038/nmeth.2089.

3. Becht, E.; Giraldo, N.A.; Lacroix, L.; Buttard, B.; Elarouci, N.; Petitprez, F.; Selves, J.; Laurent-Puig, P.; Sautès-Fridman, C.; Fridman, W.H., et al. Estimating the population abundance of tissue-infiltrating immune and stromal cell populations using gene expression. *Genome biology* **2016**, *17*, 218, doi:10.1186/s13059-016-1070-5.
