# Supplementary material for: MiR-200c-3p Contrasts PD-L1 Induction by Combinatorial Therapies and Slows Proliferation of Epithelial Ovarian Cancer through Downregulation of β-Catenin and c-Myc
Source: Cells. 2021 Mar 1;10(3):519. doi: 10.3390/cells10030519 (PMC7998372; doi:10.3390/cells10030519)
Supplement: Supplementary file 1 [file cells-10-00519-s001.zip › cells-1099740 supplementary proofreading back/Supplementary material/Table S1.docx]

**Table S1.** Description of 46 OC cell lines from Cancer Cell Line Encyclopedia (CCLE) used for the *in silico* analyses.

| **Ovarian cancer**  **cell lines identity** | **Ovarian cancer cell line**  **Subtype** | **Primary Metastasis** |
| --- | --- | --- |
| 59M_OVARY | Ovary High Grade Serous | Metastasis |
| A2780_OVARY | Ovary Endometrioid | Primary |
| CAOV3_OVARY | Ovary High Grade Serous | Primary |
| CAOV4_OVARY | Ovary High Grade Serous | Metastasis |
| COV318_OVARY | Ovary High Grade Serous | Metastasis |
| COV362_OVARY | Ovary High Grade Serous | Metastasis |
| COV434_OVARY | Ovary Endometrioid | Primary |
| COV644_OVARY | Ovary Mucinous | Primary |
| EFO21_OVARY | Ovary Clear Cell | Metastasis |
| EFO27_OVARY | Ovary Endometrioid | Metastasis |
| ES2_OVARY | Ovary Clear Cell | Primary |
| FUOV1_OVARY | Ovary High Grade Serous | Primary |
| HEYA8_OVARY | Ovary Low Grade Serous | Metastasis |
| IGROV1_OVARY | Ovary Endometrioid | Primary |
| JHOC5_OVARY | Ovary Clear Cell | Primary |
| JHOM1_OVARY | Ovary Mucinous | Primary |
| JHOM2B_OVARY | Ovary Mucinous | Primary |
| JHOS2_OVARY | Ovary High Grade Serous | Primary |
| JHOS4_OVARY | Ovary High Grade Serous | Primary |
| KURAMOCHI_OVARY | Ovary High Grade Serous | Metastasis |
| MCAS_OVARY | Ovary Mucinous | Primary |
| NIHOVCAR3_OVARY | Ovary High Grade Serous | Metastasis |
| OAW28_OVARY | Ovary High Grade Serous | Metastasis |
| OAW42_OVARY | Ovarian Cystadenocarcinoma | Metastasis |
| OC314_OVARY | Ovary Serous | Metastasis |
| ONCODG1_OVARY | Ovary High Grade Serous | Metastasis |
| OV56_OVARY | Ovary Serous | Metastasis |
| OV7_OVARY | Ovary Clear Cell | Primary |
| OV90_OVARY | Ovary High Grade Serous | Metastasis |
| OVCAR4_OVARY | Ovary High Grade Serous | Metastasis |
| OVCAR8_OVARY | Ovary High Grade Serous | Primary |
| OVISE_OVARY | Ovary Clear Cell | Metastasis |
| OVK18_OVARY | Ovary Endometrioid | Metastasis |
| OVKATE_OVARY | Ovary High Grade Serous | Primary |
| OVMANA_OVARY | Ovary Clear Cell | Primary |
| OVSAHO_OVARY | Ovary High Grade Serous | Metastasis |
| OVTOKO_OVARY | Ovary Clear Cell | Metastasis |
| RMGI_OVARY | Ovary Clear Cell | Metastasis |
| RMUGS_OVARY | Ovary Mucinous | Primary |
| SKOV3_OVARY | Ovary Serous | Metastasis |
| SNU119_OVARY | Ovary High Grade Serous | Metastasis |
| SNU840_OVARY | Brenner Tumor | Primary |
| SNU8_OVARY | Ovary High Grade Serous | Metastasis |
| TOV112D_OVARY | Ovary Endometrioid | Primary |
| TOV21G_OVARY | Ovary Clear Cell | Primary |
| TYKNU_OVARY | Ovary High Grade Serous | Primary |
